# UGT1A4 Polymorphism is not Associated with a Clinically Relevant Change in Giredestrant Exposure

**DOI:** 10.1007/s00280-023-04634-4

**Published:** 2024-02-02

**Authors:** Vikram Malhi, Malgorzata Nowicka, Ya-Chi Chen, Priya Agarwal, Marie Waldvogel, Yi Ting Kayla Lien, Marc Hafner, Pablo Perez-Moreno, Heather M. Moore, Jiajie Yu

**Affiliations:** 1 Department of Clinical Pharmacology, Genentech, Inc., South San Francisco, CA 650-484-6516 USA; 2 Oncology Biomarker Development, Genentech, Inc., South San Francisco, CA USA; 3 Product Development Clinical Operations, Genentech, Inc., South San Francisco, CA USA; 4 Oncology Bioinformatics, Genentech, Inc., South San Francisco, CA USA; 5 Product Development Oncology, Genentech, Inc., South San Francisco, CA USA

**Keywords:** UGT1A4, Polymorphism, Pharmacokinetic, SERD, Giredestrant

## Abstract

**Purpose:**

Giredestrant is a potent, orally bioavailable, small-molecule selective estrogen receptor antagonist and degrader (SERD) that is being developed for the treatment of patients with estrogen receptor (ER)-positive breast cancer. In vitro, giredestrant was primarily metabolized by UGT1A4. The goal of this study was to investigate if UGT1A4 polymorphism had a clinically relevant impact on giredestrant exposure.

**Methods:**

Genotyping and pharmacokinetic data were obtained from 118 and 61 patients in two clinical studies, GO39932 [NCT03332797] and acelERA Breast Cancer [NCT04576455], respectively.

**Results:**

The overall allelic frequencies of UGT1A4*2 and UGT1A4*3 were 3.3% and 11%, respectively. Giredestrant exposure was consistent between patients with wild-type UGT1A4 and UGT1A4*2 and *3 polymorphisms, with no clinically relevant difference observed. In addition, haplotype analysis indicated that no other UGT1A4 variants were significantly associated with giredestrant exposure.

**Conclusion:**

Therefore, this study indicates that UGT1A4 polymorphism status is unlikely a clinically relevant factor to impact giredestrant exposure and giredestrant can be administered at the same dose level regardless of patients’ UGT1A4 polymorphism status.

## Introduction

Uridine 5’-diphosphate-glucuronosyltransferases (UGTs) play a central role in catalyzing phase II glucuronidation reactions. With the addition of glucuronic acid to chemically divergent substrates, the glucuronidation process converts substrates into water-soluble glucuronides which rarely retain biological activities and are more easily eliminated from systemic circulation via renal and biliary excretion [1].

In particular, the UGT1 and UGT2 families play an important role in the metabolism of xenobiotics [2]. Although there is a remarkable redundancy between UGTs to ensure a stable glucuronidation function [1], UGT genetic variations have been reported to have a clinically significant impact on drug pharmacokinetics. For example, SN-38, the active metabolite of irinotecan, is metabolized and inactivated through glucuronidation by UGT1A1. Patients with the UGT1A1*28 or UGT1A1*6 polymorphism were reported to have reduced UGT1A1 enzyme activity, elevated SN-38 concentration, and increased risk for severe diarrhea or neutropenia following initiation of irinotecan treatment [3].

Giredestrant is a potent, orally bioavailable, small-molecule selective estrogen receptor antagonist and degrader (SERD) that is being developed for the treatment of patients with estrogen receptor (ER)-positive breast cancer. Giredestrant demonstrated a favorable pharmacokinetic profile, with adequate half-life supporting once daily (Q.D.) dosing and generally dose-proportional increase in plasma exposure in the range of 10 to 250 mg [4]. In vitro, giredestrant is primarily metabolized by UGT1A4 and to a lesser extent by CYP3A4 [data on file].

More than 100 single nucleotide polymorphisms (SNPs) have been identified for UGT1A4 [5]. The two most extensively studied polymorphisms include UGT1A4*2 (rs6755571, 70C>A, P24T) and UGT1A4*3 (rs2011425, 142T>G, L48V). Although UGT1A4*2 and UGT1A4*3 polymorphisms have been generally associated with a decrease and increase of glucuronidation activity, respectively, a consensus on the impact of these polymorphisms has not been reached [5–9]. To ensure that genetic variations do not impact the risk–benefit profile of giredestrant, we investigated whether UGT1A4 polymorphisms have a clinically relevant impact on giredestrant exposure.

## Methods

### Patient data

Genotyping and pharmacokinetic (PK) data were obtained from patients in two studies, GO39932 [NCT03332797] and acelERA Breast Cancer (BC) [NCT04576455]. Genotyping data was obtained from patients who had available whole blood samples and appropriate consent from 120 patients in GO39932 and 62 patients in acelERA BC. GO39932 is a phase Ia/Ib dose escalation/expansion study in patients with advanced or metastatic breast cancer, giredestrant doses ranged from 10 to 250 mg Q.D. Matched genotyping and PK data were included from 118 patients dosed daily with 10 mg (*n* = 5), 30 mg (*n* = 27), 90 mg (*n* = 7), 100 mg (*n* = 72), and 250 mg (*n* = 7). AcelERA BC is a phase II study in patients with advanced or metastatic breast cancer who received giredestrant 30 mg Q.D., and matched genotyping and PK data were included from 61 patients from this study.

### UGT genotyping

Genotyping data were generated using the Roche Customized Illumina GSAv3 microarray (Infinium Global Screening Array). The array types 665,732 SNP markers, including 577 markers encoding for UGT genes. Additionally, 3466 UGT SNPs were imputed using Beagle 5.1 with hg38 HapMap genetic maps and whole genome sequencing reference panels. The GSAv3 microarray included rs6755571 (UGT1A4*2); while rs2011425 (UGT1A4*3) was imputed.

### Pharmacokinetic data

Individual steady state exposure metrics for giredestrant, specifically AUC_0-24_ and C_max_, were obtained from a giredestrant population pharmacokinetic model, which was a two-compartment model with sequential zero- and first-order absorption. Except for one patient’s exposure metrics were obtained from the noncompartmental analysis due to inconsistency in the dosing record. Dose-normalized exposure metrics were used for the assessment of their relationships with the UGT1A4 genotype, given the dose-proportional PK for giredestrant.

### Data analysis

To quality control the UGT1A4 variants for possible genotype calling errors we performed the Hardy–Weinberg equilibrium test using the HWExact function from the HardyWeinberg R package. Next, for each patient and UGT1A4 variant, the genotyping results were summarized to binary values where 0 corresponds to two reference alleles and 1 to at least one alternative allele. To account for linkage disequilibrium between alleles in different loci and to reduce the number of correlated statistical tests we grouped the variants with identical genotypes (represented by binary vectors) into haplotype blocks and performed statistical testing at the block level.

Variant blocks with minor variant allele frequency (VAF) lower than 5% were filtered out. Non-parametric Kruskal–Wallis test was used to identify statistical significance between the PK exposure metrics and genotypes. Benjamini–Hochberg procedure was used to perform multiple-testing correction and associations where statistical comparisons were two-tailed and adjusted p-value of less than 0.05 was considered statistically significant. The computation of haplotype blocks, filtering, and association analysis were performed for each study separately.

## Results

UGT1A4*2 and *3 polymorphisms were found to be mutually exclusive in both clinical trials studied**.** The overall allelic frequencies of UGT1A4*2 and UGT1A4*3 were 3.3% and 11%, respectively. The majority of patients were heterozygous for *2 and *3 in both studies, with only one patient homozygous for *2 in each trial, and one patient homozoygous for *3 in acelERA BC. No patients were homozygous for *3 in GO39932.

**Fig. 1:**
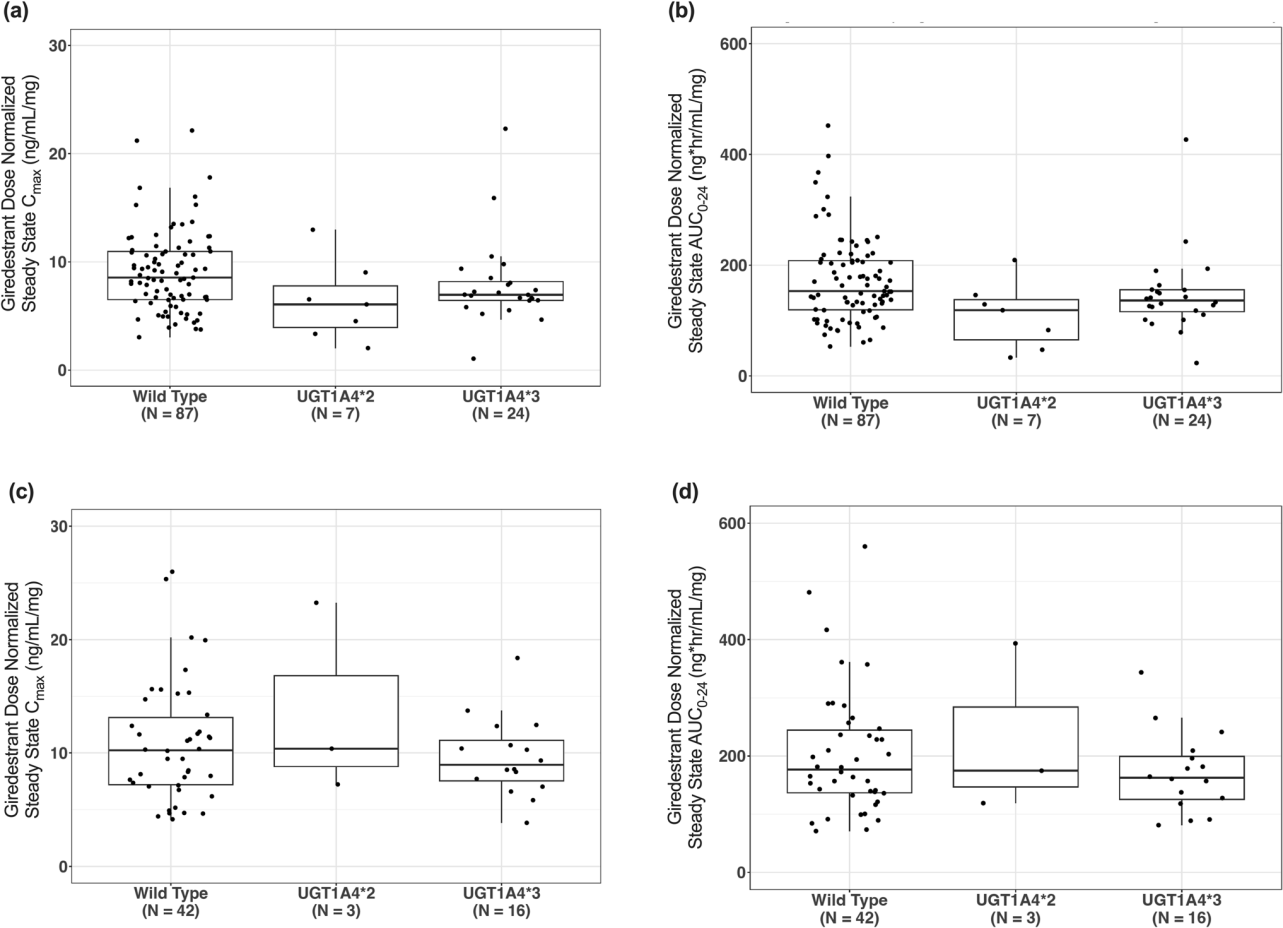
UGT Status and Dose-Normalized Steady State **A** C_max_ from GO39932 and **B** AUC_0-24_ from GO39932, **C** C_max_ from acelERA BC and **D** AUC_0-24_ from acelERA BC. The solid line in the center represents the median, the solid line at the bottom is the 25th percentile (Q1), the solid line at the top is the 75th percentile (Q3), the boxes indicate the interquartile range (IQR; Q1–Q3), and the whiskers represent 1.5*IQR

Boxplots comparing UGT status and dose-normalized steady-state C_max_ and AUC_0-24_ from GO39932 and acelERA BC are shown in Fig. 1A–D, respectively. Giredestrant PK was consistent between patients with wild-type UGT1A4 and UGT1A4*2 and *3 polymorphisms. No clinically relevant difference was observed.

In addition to UGT1A4*2 and *3, other variants of UGT1A4 with minor VAF above 5% were examined. In GO39932, variants were identified for 332 loci and were grouped into 132 haplotype blocks, among which 43 had a prevalence of higher than 5%. In acelERA BC, variants were identified for 297 loci and were grouped into 95 haplotype blocks, among which 37 had a prevalence higher than 5%. Overall, after p-value adjustment, there were no variants observed to be significantly associated with either C_max_ or AUC_0-24_ in either study.

**Fig. 2:**
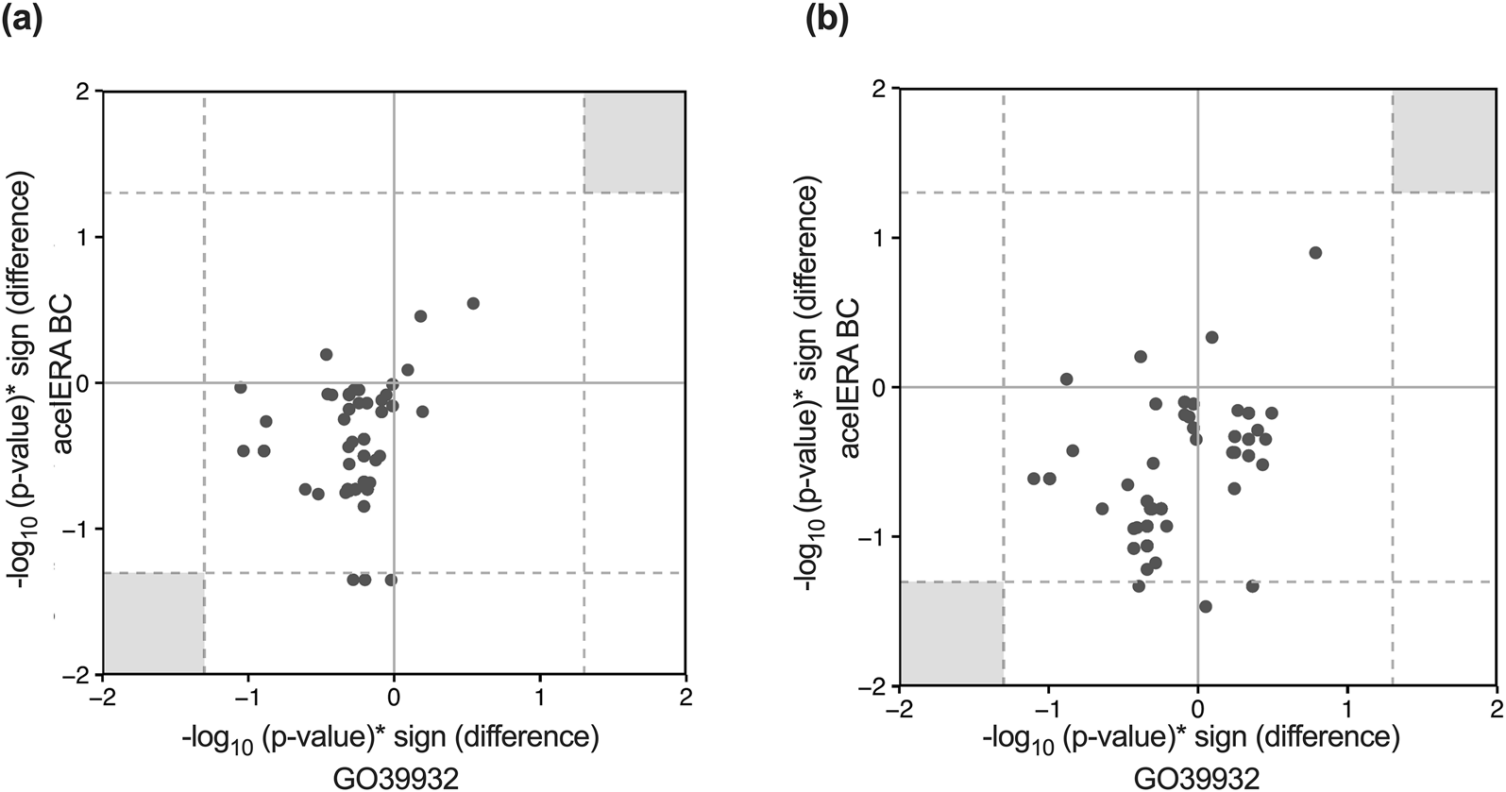
Comparison of Association Strength between UGT Variants and Steady State **A** C_max_ and **B** AUC_0-24_ from GO39932 and acelERA BC. Dashed lines indicate -log_10_ for unadjusted *p*-value = 0.05; sign is either (+) or (−); grey shading indicates where statistically significant associations (based on unadjusted p-value) that are observed in both studies would occur

To investigate if there are any variants that exhibit a similar trend for association with PK in the studies, we plotted the −log10 (*p*-value) multiplied by the sign (+ or −) of the PK difference between wild type and altered groups for each variant (Fig. 2). This statistic represents the degree and the direction of associations between PK metrics and UGT variants. However, no variants were identified to display consistent association, further supporting lack of association between UGT1A4 variants and giredestrant exposure.

## Discussion

In vitro, giredestrant is primarily metabolized by UGT1A4. Genetic variations related to phase 2 metabolizing enzymes could have a clinically relevant impact on drug exposure, as observed with irinotecan. Therefore, it was important to understand what potential liability UGT1A4 polymorphisms might have on giredestrant exposure, and more importantly would that liability be clinically relevant. One way to approach this assessment would be to evaluate the contribution of glucuronidation to the overall elimination of the drug; however, this determination in humans is not so simple for two important reasons. Glucuronides are excreted through the bile and are subject to enterohepatic recirculation; therefore, the amount of glucuronide detected in the urine and feces may not provide an accurate assessment of the contribution of glucuronidation [10]. Furthermore, the collection of bile in humans is invasive and not routinely performed.

Given the aforementioned challenges, we examined indirect evidence to shed light on the potential contribution of glucuronidation to giredestrant elimination and evaluated the findings under the clinical context. First, we compared giredestrant PK between White and Asian patients, because the frequency of UGT1A4 polymorphism has been reported to be different between racial/ethnic groups. The reported frequencies of UGT1A4*2 and UGT1A4*3 are 4.9–8.8% and 5.8–12.9% for Whites, respectively, compared to 0% and 7.4–27.0% for Asians, respectively [5–9, 11–18]. In the phase Ia/Ib dose escalation/expansion study (GO39932), no difference in giredestrant PK was observed between White and Asian patients [4]. Given the differential expression of the *2 and *3 polymorphisms in these two populations, the consistency in PK suggests the presence of these polymorphisms may not have a large effect on giredestrant PK. Second, the variability of giredestrant PK [4] is consistent with other small-molecule drugs in oncology [19]. If giredestrant PK was susceptible to large changes mediated by UGT1A4 polymorphism, higher degrees of variability would be observed. Finally, giredestrant has a relatively large therapeutic window. The clinical dose of giredestrant is 30 mg Q.D. In the phase I dose-escalation study, giredestrant was dosed up to 250 mg Q.D. with no dose-limiting toxicities or maximum tolerated dose detected. Objective response and clinical benefit were observed at all dose levels [20]. This large therapeutic window suggests that even if UGT1A4 polymorphisms were at play, it may not be clinically relevant.

Yet, the clinical relevance of UGT1A4 polymorphisms may still be inconclusive and substrate-specific. Reports are available regarding polymorphisms for several UGT1A4 substrates including lamotrigine, olanzapine, posaconazole, and tamoxifen. When UGT1A4 polymorphisms were found to impact the concentration of UGT1A4 substrates, generally changes in concentration would be consistent with reduced UGT1A4 activity for *2, and increased UGT1A4 activity for *3 [5–9, 11, 12, 15, 17]. In a few cases, these differences in activity have resulted in a statistically significant difference in serum concentration of the UGT1A4 substrate or metabolites related to UGT1A4 activity, but there are very few cases where altered UGT1A4 activity has been associated with a clinical outcome [5, 6, 8, 12, 17].

Although the indirect evidence suggested that UGT1A4 polymorphism may not be of concern for giredestrant, the availability of genotyping data enabled direct investigation of the association between UGT1A4 polymorphisms and giredestrant exposure. Giredestrant steady-state exposure (C_max_ and AUC_0-24_) was found to be consistent between patients with wild-type UGT1A4 and UGT1A4*2 and *3 polymorphisms. The allelic frequencies of patients with *2 and *3 polymorphism observed in our dataset, across both studies, are consistent with literature, indicating our dataset has a robust and diverse population that was representative of frequency expectations [5–9, 11–18]. Furthermore, no other UGT1A4 SNPs were found to be significantly associated with C_max_ or AUC_0-24_, and any association found to be close to the significance cutoff in either study (GO39932 or acelERA BC) was not consistent in the other study, further strengthening the analysis. An exploratory association analysis combining the GO39932 and acelERA BC studies was also conducted to confirm the lack of association between any UGT1A4 variants and giredestrant exposure. The results indicated UGT1A4 metabolism may not play as critical a role in giredestrant metabolism in humans as initially suggested from the in vitro findings and the risk for giredestrant having a clinically relevant drug-drug interaction as a UGT1A4 substrate may be low.

This study suggested the risk assessment for UGT1A4 polymorphisms could be conducted on a case-by-case basis and take into consideration the drug's clinical profile, consistent with the ICH M12 draft guidance recommendation. In the case of a drug with a wide therapeutic window, investigating indirect evidence may enable a certain level of de-risking. In the case of a drug with a narrow therapeutic window and/or high PK variability and when the relative in vivo contribution of glucuronidation is not known or is known to be a major route of elimination more robust assessments such as the genotyping analysis may be appropriate.

In conclusion, this study elucidates that UGT1A4 polymorphism is not associated with clinically relevant change in giredestrant exposure. Giredestrant can be administered at the same dose level to patients regardless of their UGT1A4 polymorphism status.

## Data Availability

For eligible studies, qualified researchers may request access to individual patient-level clinical data through a data request platform. At the time of writing, this request platform is Vivli: https://vivli.org/ourmember/roche/. For up-to-date details on Roche's Global Policy on the Sharing of Clinical Information and how to request access to related clinical study documents, see here: https://go.roche.com/data_sharing. Anonymized records for individual patients across more than one data source external to Roche cannot, and should not, be linked due to a potential increase in risk of patient reidentification.
